# Clinical review of how glucagon-like peptide-1 agonist obesity medications decrease sexual desire, and a biopsychosocial model for why we don’t ‘see’ it

**DOI:** 10.1016/j.obpill.2025.100233

**Published:** 2025-11-20

**Authors:** Sonya T. Gelfand, Meghan C. Tveit, James A. Simon

**Affiliations:** a George Washington University, Washington, DC, 20052, United States; b Lenox Hill Hospital, Obstetrics and Gynecology Department, New York, NY, 10075, United States; c Department of Obstetrics and Gynecology, George Washington University School of Medicine and Health Sciences, United States; d IntimMedicine Specialists, Washington, DC, 20036, United States

**Keywords:** Biopsychosocial framework, Glucagon-like peptide-1 receptor agonist medications, Obesity, Sexual desire, Serotonin, Obesity treatment

## Abstract

**Background:**

While prevailing assumptions suggest that improved body image and erectile function, even in people with diabetes, associated with glucagon-like peptide-1 (GLP-1) agonist medications would correlate with heightened sexual function, there is limited literature on the effects of GLP-1 agonists on hedonistic pleasures such as sexual activity. In this paper, we aim to elucidate the potential implications of GLP-1 agonists on sexual desire by proposing a serotonergic mechanism by which GLP-1 agonists theoretically decrease sexual desire, and a biopsychosocial perspective on why this effect may be camouflaged by competing influences.

**Methods:**

This was a narrative review analysis with target literature search. A PubMed search was conducted to identify systematic reviews or meta-analyses investigating the effects of GLP-1 agonists on physiological and lifestyle factors. We used the same methodology to investigate the connection between GLP-1 agonism and the brain’s reward pathways to elucidate whether a connection exists between GLP-1 agonism and sexual desire.

**Results:**

We established a theoretical model for how GLP-1 agonist modulation via increased serotonergic activity at the 5-HT2C receptor may result in diminished sexual desire. We then applied a biopsychosocial framework to highlight why this effect may be overlooked by GLP-1-treated patients and clinicians. Although the serotonergic pathway may create a physiological decrease in sexual desire for patients taking GLP-1 agonists, we postulate that this diminishing influence of GLP-1 agonism is both compounded by factors such as undesirable side effects and increased SHBG and offset by the enhancing influences on sexual desire such as increased total testosterone, and improved vascular reactivity and mood.

**Conclusion:**

Failing to systematically measure and report on sexual desire as a potential adverse outcome of GLP-1 agonist use overlooks an essential aspect of patient well-being. Future research should prioritize longitudinal studies to assess changes in sexual desire among individuals prescribed GLP-1 agonists.

## Graphical abstract

**Figure:**
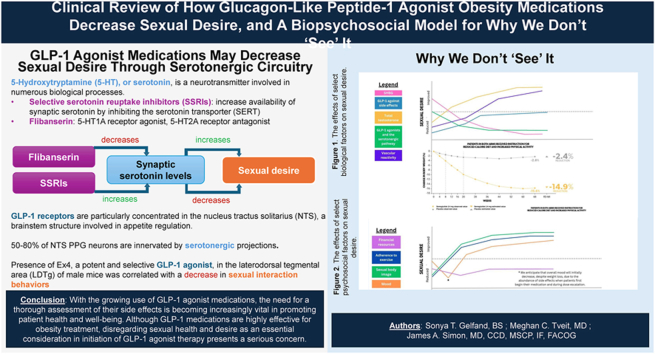

## 1 Introduction

Individuals with obesity or overweight are often excluded from the modern conception of a desirable feminine body. Weight-based discrimination has been documented across multiple settings [[1], [2], [3], [4], [5], [6], [7]], with an average 15.5 % of women reporting discriminatory experiences due to weight/height in 2004–2006, a number that increased from 10 % in 1995–1996 and has continued to rise [8]. Driven in part by this stigmatization, recent efforts have revolved around developing obesity medications to help individuals achieve a desired body image and maintain better health. Glucagon-like peptide-1 (GLP-1) agonists are an example of such a medication, garnering remarkable traction for their role in treating type II diabetes and inducing weight loss. While prevailing assumptions suggest that improved body image [9] and erectile function associated with GLP-1 agonism, even in people with diabetes [[10], [11], [12]], would correlate with heightened sexual desire and function, limited research has been done on the clinical outcomes and implications of GLP-1 agonism on hedonistic pleasures such as sex in men or women. Emerging literature suggests that like their ability to reduce desire for food, GLP-1 agonists can also reduce desire for other hedonistic pleasures such as cannabis [13,14], cigarettes [13], alcohol [13], opiates [15,16], and sexual desire [17] in patients with obesity or diabetes. Similarly, a recent case report documented an association between GLP-1 agonist therapy and sexual dysfunction in a patient with overweight [18]. In this paper, we explore a hypothesis that GLP-1 agonism leads to a marked decrease in sexual desire, with other confounding influences camouflaging that decrease. This assertion stems from a biopsychosocial framework [19], i.e., that a GLP-1 agonist-associated reduction in appetite for food may be associated with a diminished desire for all hedonistic activities, including sex, a biological assumption rooted in serotonergic neural circuitry. Here, we provide a narrative review analysis with target literature search conducted through a theoretical framework to explicate the relationship between GLP-1 agonism and sexual desire.

## 2 A theoretical model – the serotonin pathway

GLP-1 agonists are becoming increasingly significant in the treatment of diseases like diabetes, metabolic syndrome, and obesity, mimicking the action of endogenous GLP-1, an anorectic hormone produced in multiple human body tissues including the intestine, pancreas, and central nervous system [20,21]. GLP-1 regulates feeding behavior, glycemic control, and stress [21]. Mechanistically, GLP-1 enhances insulin secretion in response to food intake, inhibits glucagon release, and slows gastric emptying, contributing to its therapeutic role in reducing blood glucose levels and body weight [21].

While a majority of GLP-1 is synthesized in the gut through posttranslational processing of the proglucagon gene in enteroendocrine L-cells [21], the central nervous system plays a smaller, and often less appreciated role in endogenous GLP-1 production. In the central nervous system, GLP-1 is produced by preproglucagon (PPG) neurons located in the caudal nucleus of the solitary tract, projecting to the hypothalamus, forebrain, hindbrain, and mesolimbic brain regions [22]. These regions are known to control feeding behaviors, including hunger-driven feeding, motivation, and the hedonic value of food [23].

### 2.1 Serotonin and sexual desire

5-Hydroxytryptamine (5-HT), or serotonin, is a monoamine neurotransmitter that contributes to the regulation of mood, appetite, sleep, and other physiological functions through several different 5-HT receptors. Increased activity at the 5-HT1A receptor is linked to mood regulation, often being associated with anxiolytic and antidepressant effects. Overactivation at the 5-HT2A receptors can lead to side effects such as insomnia, agitation, or sexual dysfunction, and modulation of the 5-HTC receptor family can influence appetite and weight changes. In the context of sexual behavior and desire, serotonin seems to have a somewhat dual role. On one hand, selective serotonin reuptake inhibitors (SSRIs) – typically indicated for the treatment of depression and anxiety due to their ability to increase synaptic serotonin levels – have a known side effect of anorgasmia and decreased sexual desire [24]. In contrast, flibanserin – a dual acting 5-HT1A receptor agonist and 5-HT2A receptor antagonist developed to treat hypoactive sexual desire disorder (HSDD) in premenopausal women – is associated with increased sexual desire [19,25,26]. It thus becomes clearer that serotonin may play a key role in modulating sexual desire: an increase in synaptic serotonin may be associated with dampened sexual desire, while a decrease in specific serotonin receptor activity may be associated with enhanced sexual desire.

### 2.2 Serotonin and GLP-1 agonists

While the interaction between GLP-1 and serotonin within the central nervous system is an emerging area of research, it is known that GLP-1 receptors are particularly concentrated in the nucleus tractus solitarius (NTS), a structure in the brainstem responsible for key homeostatic functions, including appetite regulation. Holt et al. determined that 50–80 % of NTS PPG neurons are innervated by serotonergic projections [27]. Furthermore, rodent models have shown that central blockade of GLP-1 receptor signaling decreases the appetite-suppressing effects of hindbrain serotonin [28]. Conversely, activation of hindbrain 5-HT2C receptor acutely suppresses feeding in a GLP-1 dependent manner [28]. Holt et al. also found that the food intake-suppressing effect of GLP-1 administered intraperitoneally was abolished in mice lacking 5-HT2C receptors, further highlighting the role of the 5-HT2C receptor in the modulation of GLP-1 effects [27].

Our theoretical model thus proposes that GLP-1 agonism and serotonin activity are closely linked. An increase in serotonergic 5-HT2C receptor activity seemingly increases effects of GLP-1 agonism, and increased synaptic serotonin is simultaneously associated with decreased sexual response and desire. We therefore suggest that through serotonergic circuitry, GLP-1 agonism may indeed be associated with a decrease in sexual desire and behavior.

### 2.3 GLP-1 agonists and sexual behavior

In a prior publication, we highlighted an analysis by Vestlund and Jerlhag of sexual behavior in male mice which showed that the presence of Ex4, a potent and selective GLP-1 agonist, in the laterodorsal tegmental area (LDTg) was correlated with a decrease in sexual interaction behaviors [29,30]. Administration of Ex4 into the posterior ventral tegmental area (pVTA) was similarly associated with decreased sexual behavior. Because LDTg and pVTA are reward-driven pathways in the brain, the influence of Ex4 on these brain regions provides valuable insight into the action of GLP-1 agonists on reward-driven behaviors, including sexual pursuits [30]. These findings thus underscore the intimate inverse relationship between GLP-1 agonism and sexual pursuits, guided by libido/sexual desire: as GLP-1 agonism increases, sexual pursuits decrease.

Although the physiological evidence suggests an inverse relationship between GLP-1 agonism and sexual desire, we posit that this relationship is difficult to observe in clinical practice, particularly due to the competing factors influencing sexual desire in patients undergoing obesity treatment through the use of GLP-1 agonists. In the next section, we take a biopsychosocial approach to elucidate why this may be the case.

## 3 The biological perspective

**Fig. 1:**
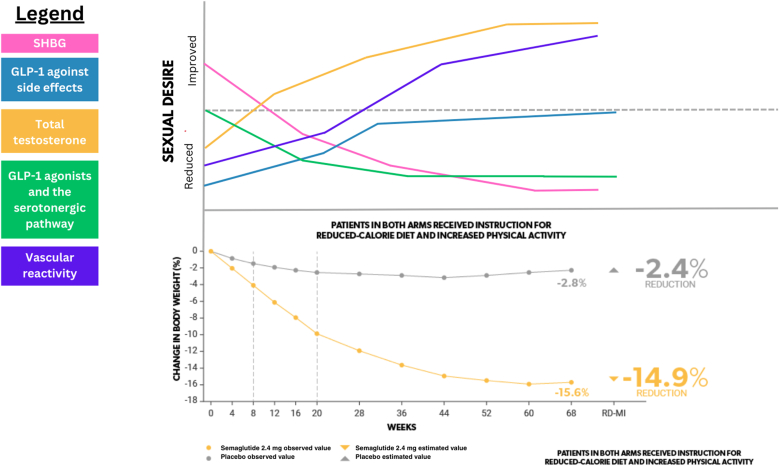
Biological factors contributing to sexual desire in patients taking semaglutide 2.4 mg.

In addition to the impact of GLP-1 agonists on the serotonin system, we posit that the biological influences of GLP-1 agonists on individuals’ sexual desire are multifaceted and often competing. In this section, we introduce a few key effects that we believe modulate this relationship, using semaglutide 2.4 mg as a model for GLP-1 agonists. To better illustrate the multifaceted nature of semaglutide-associated biological influences on sexual desire, we use Fig. 1 to postulate how these influences mediate a change in sexual desire from baseline. The semaglutide 2.4 mg (Wegovy) obesity treatment data [31] is presented alongside the graph to better illustrate how these biological influences may impact obesity treatment progression. Forecasted changes in sexual desire illustrated in Fig. 1 are not rooted in quantitative evidence, but rather present an estimated assessment based on the data provided below. For additional effects not included in Fig. 1, see Table 1.

**Table 1:** Factors modulating GLP-1 agonist-associated changes in sexual desire.

Factors Diminishing Sexual Desire	Factors Enhancing Sexual Desire
Weight-based discrimination [[1], [2], [3], [4], [5], [6], [7], [8]]	Improved body image [9,46]
Modulation of the serotonin pathway [[27], [28], [29], [30]]	Reduced use of alcohol, cannabis, tobacco, and opiates [[13], [14], [15], [16]]
Gastrointestinal side effects [31,70]	Enhanced glycemic control [21]
Increased SHBG levels with reduced free testosterone [[40], [41], [42], [43]]	Increased total testosterone [38,39]
Persistent lack of confidence in body image [47]	Improved systemic blood flow [46]
Diminished financial flexibility [58,59]	Improved mood and sense of well-being [53,54]
Aesthetic side effects such as “Ozempic face” [67]	Increased physical activity and adherence to exercise [56,57]
Fatigue [68]	Improved sleep [65,66]
Weight regain [69]	Improved erectile function and female arousal response [[10], [11], [12],^72^]
Stigmatizing attitudes towards obesity treatment with GLP-1 agonists [71]	Improved perceived societal acceptance [73]

**Note:** Content not meant to be all inclusive.

### 3.1 Testosterone

For most women, testosterone levels tend to surge at the time of ovulation, known to be a period of heightened sexual desire in the menstrual cycle [32]. Research surrounding testosterone administration in women with hypoactive sexual desire has supported the enhancing effect of testosterone on sexual desire [33]. Adipose tissue, a target of GLP-1 agonists, plays a key role in testosterone production. Adipose tissue can store and metabolize estrogens, deriving from the presence of aromatase in adipose tissues and high concentrations of adrenal androgens throughout life [34,35]. Therefore, an increased quantity of adipose tissue in individuals with obesity creates an increase in estrogen concentrations, primarily estrone. Estrogens, in turn, have negative feedback effects on the hypothalamic-pituitary-gonadal axis, first lowering gonadotropin-releasing hormone (GnRH), which in turn lowers both gonadotropins, follicle stimulating hormone (FSH) and luteinizing hormone (LH) [36]. Ultimately, these decreases reduce stimulation of the testes and ovaries to produce testosterone [37], a mediator of – among other effects – sexual desire. So, we predict that in the obese state, adipose tissue derived estrogens reduce gonadal testosterone and subsequent sexual desire. We thus anticipate that semaglutide-associated weight loss – and thus loss of adipose tissue – would therefore have the opposite effect: an increase in total testosterone and sexual desire [38,39], as illustrated by the yellow line in Fig. 1.

### 3.2 SHBG

Studies have suggested that obesity treatment increases SHBG levels from low levels in the obese and overweight state in patients with polycystic ovarian syndrome (PCOS) [40,41]. Physiologically, SHBG has a role in limiting the bioavailability of free sex hormones, including testosterone [42]. Therefore, we hypothesize that an increase in SHBG during obesity treatment [43] will contribute to a decrease in sexual desire by increasing bound testosterone compared to free testosterone. This predicted trend is visualized by the pink line in Fig. 1.

### 3.3 GLP-1 agonist side effects

GLP-1 agonists present several unfavorable side effects for patients. Studies have reported that gastrointestinal disturbances such as nausea, vomiting, diarrhea, and constipation are common (incidence rate 44 %, 30 %, 24 %, and 24 %, respectively [31]) and often dose- and time-dependent in patients with overweight or obesity [41,44,45]. Therefore, we hypothesize that upon initiation of GLP-1 agonist therapy and dose escalation, patients will have decreased sexual desire due to side effect-associated discomfort. As these side effects subside over time, we predict that sexual desire will improve accordingly. The predicted steady increase in sexual desire overtime due to GLP-1 agonist-associated side effects is illustrated by the blue line in Fig. 1.

### 3.4 Vascular reactivity

Studies have suggested that GLP-1 based therapies increase vascular nitric oxide production, thus improving systemic blood flow [46]. We postulate that this improvement will increase vascular reactivity in the arousable tissues of the genitals (i.e. clitoris, penis), increasing sexual arousal, orgasm, and secondarily by reinforcement and reward, sexual desire, as predicted by the purple line in Fig. 1.

## 4 The psychosocial perspective

**Fig. 2:**
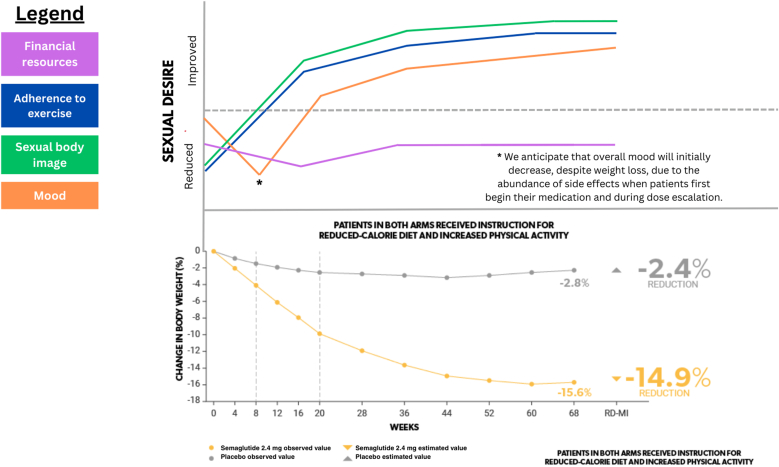
Psychosocial factors contributing to sexual desire in patients taking semaglutide 2.4 mg.

It is likely that there are also several psychosocial factors contributing to changes in sexual desire for patients losing weight on GLP-1 agonists. Here, we introduce several factors potentially modulating this relationship, again using semaglutide 2.4 mg as a model. Fig. 2 has been constructed in the same manner as Fig. 1 with similar interpretive limitations, as described in the previous section. For additional effects not included in Fig. 2, see Table 1.

### 4.1 Sexual body image

Sexual body image is an important determinant of sexual desire. Participants in one study expressing dissatisfaction with their weight demonstrated self-consciousness and a negative body image, adversely affecting sexual satisfaction [47]. Similarly, a study found that after weight loss surgery, patients show a significant increase in sex life quality over time, discovering that the correlation between body mass index (BMI) and sex life was modulated by the influence of BMI on body dissatisfaction [46]. This suggests a positive correlation between sexual body image and sexual desire. This positive correlation is depicted by the green line in Fig. 2. We predict a decrease in the rate of change coinciding with a plateau in weight loss.

### 4.2 Mood

The literature regarding psychiatric adverse effects (AEs) of GLP-1 agonists is inconclusive; one study found no difference in depression between pharmacologic interventions for weight loss and placebo. Another study found that psychiatric AEs only accounted for 1.2 % of total reports for semaglutide, liraglutide, and tirzepatide [47,48], although rare case reports have suggested suicide as a serious psychiatric AE of semaglutide 2.4 mg [[49], [50], [51], [52]]. A study using mouse models to investigate depression linked to type 2 diabetes found that administration of semaglutide effectively decreased depressive and anxious behaviors [53], and additional studies on human subjects have made similar observations [54]. Therefore, it is difficult to predict a clear influence of semaglutide on mood. However, we anticipate that, following the alleviation of unpleasant side effects, there will be a net increase in mood due to greater self-esteem and thus an associated improvement in sexual desire. This predicted trend in sexual desire mediated by mood is illustrated by the orange line in Fig. 2.

### 4.3 Adherence to exercise

Often, individuals with obesity feel embarrassment surrounding exercising in public spaces due to fear of being viewed poorly compared to those they believe are more fit, attractive, or competent [50]. Studies have supported the assertion that these fears may result in exercise avoidance among people with obesity or overweight [55]. As individuals on semaglutide lose weight, we predict that they will likely be less fearful of such judgement and thus feel more compelled to exercise. Studies suggest that exercise indirectly improves sexual function and libido, therefore providing foundation for the predicted relationship – as illustrated by the blue line in Fig. 2 – between GLP-1 agonists and sexual desire [56,57]. We predict that, like the influence of sexual body image on sexual desire, the change in sexual desire due to exercise adherence will decrease as weight loss rate plateaus.

### 4.4 Financial resources

Financial resources likely also help mediate sexual desire in individuals taking GLP-1 agonist medications. Current insurance coverage for all obesity medications including the GLP-1 agonists is spotty yet steadily improving. In 2024, the monthly out of pocket cost of GLP-1 receptor agonists for those without insurance was over $900 [58,59]. It can be hypothesized that insufficient insurance coverage and high out of pocket costs may leave people taking GLP-1 agonists with fewer financial resources for other expenses. For example, they may sacrifice dates or social events and outings – potential mechanisms by which individuals can meet prospective sexual partners. We predict that decreased financial flexibility may also limit individuals’ ability to have flexible work schedules, thus decreasing availability to engage in sexual behavior. However, a cost-effectiveness analysis of semaglutide 2.4 mg found that semaglutide was a cost-effective treatment option for adults with obesity in the United States [60]. As depicted by the purple line in Fig. 2, we therefore anticipate that the overall influence of financial factors related to semaglutide cost will be minor, despite an initial decrease in sexual desire due to an adjustment to the added expense of GLP-1 agonist medication.

## 5 Putting it together: Why patients and practitioners don’t “see” the adverse effects of GLP-1 agonists on sexual desire

In exploring the influence that GLP-1 agonists have on sexual desire through a biopsychosocial framework, it becomes clear that there are likely several competing contributing factors, and our short list is far from exhaustive. Due to the antagonistic influence of these factors on sexual desire, it is difficult to determine a “net” effect of GLP-1 agonists on sexual desire. The influence of one factor may offset that of another, creating the appearance of a camouflaged effect on sexual desire from baseline in patients taking these medications. While we have explored the mechanism by which the serotonergic pathway may create a physiological decrease in sexual desire for patients taking GLP-1 agonists, the biopsychosocial factors discussed propose a potential mechanism by which these adverse effects are masked, if not overcome. In other words, we predict that the diminishing influence of GLP-1 agonists’ use of the serotonergic pathway on sexual desire may be compounded by the diminishing influences of GLP-1 agonists such as undesirable side effects and increased SHBG, but offset by the enhancing influence of GLP-1 agonists on sexual desire such as increased total testosterone and vascular reactivity, as well as improved body image and mood. It is also important to consider the unique individual responses to weight loss and its indirect effects among those taking GLP-1 agonists [61]. The way in which these factors manifest and subsequently influence sexual desire varies between individuals due to physiological and cultural differences, thus contributing to the difficulty of observing a distinct “net” effect of GLP-1 agonists on sexual desire.

## 6 Conclusion and future directions

With the growing use of GLP-1 agonist medications (currently estimated at one in eight in the US, and one in five women aged 50–64 [62]), the need for a thorough assessment of their side effects is becoming increasingly vital in promoting patient health and well-being. Although GLP-1 medications are highly effective for obesity treatment, disregarding sexual health and desire as an essential consideration in initiation of GLP-1 agonist therapy presents a serious concern. Our biological framework proposes a potential mechanism by which serotonergic circuitry may underlie an association between GLP-1 agonist medications and a decrease in sexual desire. As evidenced by our predictions in Fig. 1, Fig. 2**,** there may also be important timing-related considerations to make when empirically investigating the influence of GLP-1 agonists on sexual desire. Our biopsychosocial approach predicts that there may be an initial decreased desire from these medications, due to a direct effect on the 5-HT pathway, GI side effects, and other factors compounding an already low baseline sexual desire based on body image. As weight loss progresses and plateaus, modulation of side effects as well as vascular and total testosterone improvements may have overall enhancing effects on sexual function. There has been a gradual emergence of research assessing sexual side effects of GLP-1 agonist medications, including the use of the Female Sexual Function Index (FSFI) at various time points following initiation of GLP-1 agonist therapy [63]. Future research should continue to prioritize longitudinal studies to monitor changes in sexual desire among individuals on GLP-1 agonist therapy. Furthermore, it may be worthwhile to explore the differential modulation of sexual desire by serotonin in contrast with dopamine given the recently documented difference in the association between each neurotransmitter and various reward processes in humans [64]. In targeting these research priorities, clinicians will be better adept at optimizing patient care and providing a comprehensive overview to patients of the side effects of GLP-1 agonist medications.

## 7 Clinical takeaways

• Our biological framework proposes a potential mechanism by which serotonergic circuitry may underlie an association between GLP-1 agonist medications and a decrease in sexual desire.
• Although physiological evidence suggests an inverse relationship between GLP-1 agonism and sexual desire, we posit that this relationship is difficult to observe in clinical practice due to the competing factors influencing sexual desire in patients undergoing obesity treatment with GLP-1 agonists. The influence of one factor may offset that of another, creating the appearance of a camouflaged — or net neutral — effect on sexual desire from baseline in patients taking these medications.
• Elucidating the relationship between GLP-1 agonists and sexual behavior is essential in developing a comprehensive understanding of GLP-1 agonist side effects and maintaining patient wellbeing.

## CRediT author statement

The concept of the submission was by James A. Simon. Writing, reviewing, and editing was done by Sonya T. Gelfand, Meghan C. Tveit, and James A. Simon. All authors reviewed, edited, and approved the final submission.

## Ethics review

Not applicable.

## Disclosures

None.

## Declaration of artificial intelligence (AI) and AI-assisted technologies utilized in the writing process

None.

## Source of funding

This manuscript was submitted without funding support.
